# Antibacterial activity of soil-isolated *Bacillus altitudinis/pumilus* complex against methicillin-resistant *Staphylococcus aureus* from Mwanza, Tanzania

**DOI:** 10.4102/ajlm.v12i1.2167

**Published:** 2023-07-18

**Authors:** Reuben N. Abednego, Vitus Silago

**Affiliations:** 1 Microbiology Laboratory, National Public Health Laboratory, Dar es Salaam, United Republic of Tanzania; 2 Department of Microbiology and Immunology, Weill Bugando School of Medicine, Catholic University of Health and Allied Sciences, Mwanza, United Republic of Tanzania

**Keywords:** *Bacillus altitudinis/pumilus* complex, Gram-positive cocci, methicillin-resistant *Staphylococcus aureus*, discovery of new antibiotic agent, soil bacteria

## Abstract

Antimicrobial resistance in methicillin-resistant *Staphylococcus aureus* and beta-lactamase-producing Gram-negative bacteria is a global health concern necessitating research and the development of effective antimicrobial agents. This study, conducted in May 2020 in Mwanza, Tanzania, aimed to determine the antibacterial activity of metabolites from soil-isolated *Bacillus* species against clinical bacterial pathogens. One soil-isolated *Bacillus* species, identified as *Bacillus altitudinis/pumilus* complex, showed antibacterial activity against Gram-positive cocci, including a methicillin-resistant *S. aureus* strain with inducible clindamycin resistance, previously isolated from a patient with osteomyelitis. *Bacillus altitudinis/pumilus* complex metabolites may be a potential source of antimicrobial agents against multidrug-resistant bacteria.

**What this study adds:**

The study supports existing research on the discovery and development of new antimicrobial agents against multi-drug-resistant bacteria. We report the antimicrobial activity of metabolites extracted from soil-isolated *Bacillus altitudinis/pumilus* complex strains against Gram-positive bacteria, including a methicillin-resistant *Staphylococcus aureus* strain with inducible clindamycin resistance.

## Introduction

The upsurge of multidrug-resistant (MDR) infections has led to an increase in healthcare costs and mortalities. Other negative impacts of MDR infections include prolonged days of hospitalisation and lack of prophylactic protection.^1,2^ Extended-spectrum beta-lactamase-producing Gram-negative bacteria, methicillin-resistant *Staphylococcus aureus* (MRSA) and inducible clindamycin-resistant *S. aureus* are common MDR bacteria reported globally.^3,4,5^ According to estimates, by 2050, MDR infections could cost up to $100 trillion United States dollars annually and cause approximately 10 million deaths per year.^6,7^ Moreover, a predictive statistical modelling study published in 2022 estimated that 1.27 million deaths were directly attributable to antimicrobial resistance (AMR), and 4.95 million deaths were associated with AMR globally in 2019.^8^ These deaths were largely due to MDR strains of *Escherichia coli, S. aureus*, and *Klebsiella pneumoniae*.^8^ These projections have led to urgent calls by the World Health Organization to guide and promote research and development of new effective antimicrobials against MDR strains.^9^ Since 1987 to date, no new class of antibiotics has been discovered and successfully introduced for clinical use, particularly for the treatment of infections caused by MDR bacteria such as extended-spectrum beta-lactamase-producing Gram-negative bacteria, MRSA and inducible clindamycin-resistant *S. aureus* strains.^10^

Currently, scientists are struggling to discover potential sources of antibiotics with activity against MDR bacteria. Various potential sources have been explored, including reptile blood,^11^ soil microorganisms,^12^ marine microorganisms,^13^ and plants.^14^ Certain microorganisms produce antimicrobial compounds such as antibiotics, antifungals, and antivirals as a means of gaining a competitive advantage in their environment where resources such as food are limited.^15^ The majority of antibiotics in clinical use today were sourced from microorganisms.^16^ Antimicrobials such as penicillin G (sourced from *Penicillium notatum*), vancomycin (*Amycolatopsis orientalis*), gentamicin (*Micromonospora purpurea*), and lincosamides (*Streptomyces lincolnensis*) are some examples that have been derived from microorganisms.^16^

This study aimed to test the antibacterial activity of *Bacillus* species isolated from soil samples in Mwanza, Tanzania, against medically important pathogenic bacteria, including MDR strains such as extended-spectrum beta-lactamase-producing Gram-negative bacteria, MRSA, and inducible clindamycin-resistant *S. aureus* isolated from clinical samples.

## Methods

### Ethical considerations

This study was approved by the joint Catholic University of Health and Allied Sciences and Bugando Medical Centre Research Ethics and Review Committee with research clearance certificate number: CREC/298/2023. The current study used bacteria species previously isolated from clinical samples at Bugando Medical Centre and stored at –80 °C in the Microbiology Laboratory at Catholic University of Health and Allied Sciences in Mwanza, Tanzania. Participants’ informed consent forms were not applicable because archived bacteria were used. Moreover, patient-related information such as socio-demographic data were not used in the current study, to ensure data confidentiality.

### Study design, duration, sampling and setting

This was a laboratory-based study conducted in May 2020 in Mwanza, Tanzania. Surface soil samples were collected in sterile Falcon™ 50 mL conical tubes (Corning, Glendale, Arizona, United States) from 20 different locations of the same site at Bugando Hill in Mwanza, Tanzania. Soil samples were sent to the Microbiology Laboratory at the Catholic University of Health and Allied Sciences and processed within 1 h of collection.

### Isolation of *Bacillus* species from soil samples

Soil samples were serially diluted in 0.85% sterile saline as described previously^17^ before being inoculated onto 5% sheep blood agar (BA; HiMedia, Mumbai, India) plates. Briefly, 1 g of soil sample was suspended in 5 mL of sterile saline, which was then vortexed for 15 s and allowed to settle for 5 min. One millilitre of the resulting supernatant was mixed with 4 mL of sterile saline, vortexed for 15 s, and then allowed to settle for 5 min. This step was repeated three times to obtain five-fold serial dilutions. From the fifth dilution, 1 mL of supernatant was inoculated on a BA plate using the pour plate technique. Plates were incubated aerobically at 37 °C for 20 h. Presumptive *Bacillus* species colonies (large-sized, dry, flat, greyish to whitish, and beta-haemolytic colonies) were selected and sub-cultured on fresh BA plates to obtain pure colonies. These plates were incubated aerobically at 37 °C for 20 h. Gram staining was used for the preliminary identification of *Bacillus* species before further analysis.

### Extraction of bacterial metabolites and antibacterial activity testing

A loopful (10 µL) of each presumptive *Bacillus* isolate was suspended in 1 mL of sterile nuclease-free water in an Eppendorf tube (1.5 mL safe lock microcentrifuge tube; Sigma-Aldrich Chemie GmbH, Taufkirchen, Germany). The tubes were incubated at 50 °C in a heating block for 30 min with intermittent vortex-mixing for 15 s every 10 min. We selected 50 °C for the extraction of bacterial metabolites because this temperature is insufficient for the destruction of metabolites. After incubation, the tubes were centrifuged at 12 000 revolutions per minute for 10 min, after which 0.8 mL of the supernatant from each tube was transferred into sterile Eppendorf tubes. The supernatants containing metabolites from soil-isolated presumptive *Bacillus* species were used immediately for antibacterial activity determination.

The well diffusion method, as described by Yilmaz and colleagues,^18^ was used to test the antibacterial activity of the extracted metabolites against one isolate each of extended-spectrum beta-lactamase-producing and non-producing *E. coli* and *K. pneumoniae*, MRSA and methicillin-susceptible *S. aureu*s, inducible clindamycin-resistant *S. aureus*, coagulase-negative staphylococci, and *Streptococcus pyogenes*. These bacterial pathogens were isolated from clinical samples of previous studies conducted in the same setting^19,20^ and archived at –80 °C in 20% glycerol stocks. The recovery of these bacterial strains was performed by subculture on BA plates which were incubated at 37 °C for 24 h. Bacterial suspensions of these test strains were prepared using sterile saline and adjusted to 0.5 McFarland standard solution (Remel, Lenexa, Kansas, United States). The bacterial suspensions were then inoculated onto the surface of Mueller Hinton agar (MHA; HiMedia, Mumbai, India) plates. Within 15 min of inoculation, wells of 6 mm in diameter were created in the inoculated plates using a cork borer (Sigma Aldrich, Darmstadt, Germany), and 100 µL of the suspension containing the extracted metabolites was then pipetted into the bored wells. The inoculated MHA plates were incubated in an upright position in ambient air at 37 °C for 20 h. The diameters of zones of inhibitions were measured in millimetres from the edge of the inhibition zone to the margin of *Bacillus* species metabolites. The complete absence of inhibition was referred to as ‘negative antibacterial activity’ and the presence of a clear zone of inhibition was referred to as ‘positive antibacterial activity’. This experiment was performed in duplicate to ensure the validity of our results. In cases of positive antibacterial activity, an average zone of inhibition was calculated and recorded as the final result.

### Identification of *Bacillus* species with positive antibacterial activity

Only one soil-isolated presumptive *Bacillus* species with a clear zone of inhibition (‘positive antibacterial activity’) was further identified at the species level. We first conducted biochemical identification tests, including catalase production, coagulase production, oxidase production, urease production, DNase production, indole production, citrate utilisation, lactose fermentation, hydrolysis of bile aesculin, and motility tests. We also tested the capacity of the isolates to grow on BA supplemented with 7% NaCl, as well as at higher incubation temperatures of 50 °C and 70 °C. The biochemical identification tests were not conclusive. For further species identification, we also used the VITEK MS (BioMérieux, Baden, Germany) system, which is an automated microbial identification system that uses the Matrix Assisted Laser Desorption Ionization Time-of-Flight technology. The latter was performed at the Microbiology Laboratory, National Public Health Laboratory in Dar es Salaam, Tanzania.

### Data analysis

Laboratory data were documented and analysed using Microsoft Excel (Microsoft Corporation, Redmond, Washington, United States). Categorical data were expressed as percentages.

## Results

**FIGURE 1:**
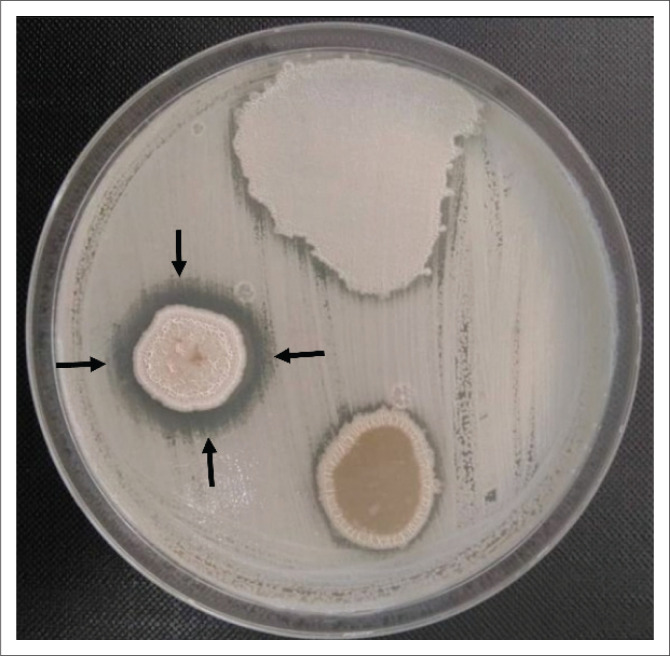
Inhibition of methicillin-resistant *Staphylococcus aureus* by *Bacillus altitudinis/pumilus* complex isolated from soil in Mwanza, Tanzania, May 2020. The zone of growth inhibition is indicated with black arrows.

A total of 20 soil samples were collected and processed for the isolation of *Bacillus* species. Out of the 20 soil samples processed, 16 (80.0%) showed growth of presumptive *Bacillus* species. Of the 16 presumptive *Bacillus* species isolated, only one (6.3%) isolate – S9D – showed antibacterial activity against Gram-positive bacteria only, including the MRSA strain, which was also inducible clindamycin-resistant (Table 1 and Figure 1).

**TABLE 1:** Antibacterial activity of *Bacillus* species isolates from soil samples against clinical bacterial isolates in Mwanza, Tanzania, May 2020.

Isolates	Gram-negative bacteria				Gram-positive bacteria			
	*Escherichia coli*		*Klebsiella pneumoniae*		*Staphylococcus* species			*Streptococcus* species
	ESBL	Non-ESBL	ESBL	Non-ESBL	MRSA + iCLI-R	MSSA	CoNS	*Streptococcus pyogenes*
S6A	-	-	-	-	-	-	-	-
S6B	-	-	-	-	-	-	-	-
S7A	-	-	-	-	-	-	-	-
S7B	-	-	-	-	-	-	-	-
S7C	-	-	-	-	-	-	-	-
S7D	-	-	-	-	-	-	-	-
S8A	-	-	-	-	-	-	-	-
S8B	-	-	-	-	-	-	-	-
S8C	-	-	-	-	-	-	-	-
S8D	-	-	-	-	-	-	-	-
S8E	-	-	-	-	-	-	-	-
S8F	-	-	-	-	-	-	-	-
S9A	-	-	-	-	-	-	-	-
S9B	-	-	-	-	-	-	-	-
S9C	-	-	-	-	-	-	-	-
S9D	-	-	-	-	2.5 mm	4.0 mm	3.5 mm	3.0 mm

CoNS, coagulase-negative Staphylococci; ESBL, extended-spectrum beta-lactamase; iCLI-R, inducible clindamycin resistance; MRSA, methicillin-resistant *Staphylococcus aureus*; MSSA, methicillin-susceptible *Staphylococcus aureus*.

-, no zone of inhibition.

The presumptive *Bacillus* species isolate with positive antibacterial activity was Gram-positive and rod-shaped with central spores on microscopic examination after Gram staining. The isolate was also β-haemolytic on 5% sheep BA and demonstrated catalase production, sugar fermentation, growth on BA supplemented with 7% NaCl, and growth at 50 °C (Table 2). Using VITEK MS, the isolate was identified as *Bacillus altitudinis/pumilus* complex with a confidence level of 99.9%.

**TABLE 2:** Biochemical characteristics of a soil-isolated *Bacillus altitudinis/pumilus* complex isolate with positive antibacterial activity against Gram-positive bacteria isolated from clinical samples, Mwanza, Tanzania, May 2020.

Biochemical identification test	Result
Haemolysis on BA	β-haemolysis
Catalase production	Positive
Oxidase production	Weak positive
Lactose fermentation	Positive
Indole production	Negative
Motility	Negative
Hydrolysis of bile aesculin	Negative
Urease production	Positive
Citrate utilisation	Negative
DNase production	Weak positive
Growth on BA containing 7% NaCl	Positive
Growth at 50 °C	Positive
Growth at 70 °C	Negative
Position of spore on bacterial cell	Centre

BA, blood agar; NaCl, sodium chloride.

## Discussion

In this study, we report a *Bacillus altitudinis/pumilus* complex isolate with antimicrobial activity against clinical Gram-positive bacterial strains, including an MRSA strain that was also inducible clindamycin-resistant. Due to the presence of several closely related species in the *Bacillus* genus, the VITEK MS system used in this study could not identify the *Bacillus altitudinis/pumilus* complex isolate at the species level.^21^ The study finding is similar to a previous study in Brazil in 2020,^22^ where marine-isolated *Bacillus altitudinis/pumilus* was shown to possess antimicrobial activity against MDR bacterial strains. Similarly, another study in Thailand in 2007 also reported that a soil-isolated *Bacillus pumilus* showed antibacterial activity against Gram-positive bacteria, including MRSA and vancomycin-resistant *Enterococcus faecalis*.^23^ The potential antimicrobial activity of *Bacillus altitudinis/pumilus* complex isolates has been linked to the production of bacilysins and bacteriocins.^22^ The report from Thailand in 2007 documented that pumilicin 4 is a bacteriocin produced by a *Bacillus pumilus* strain and had bactericidal activity against MRSA strains.^23^

We observed no antibacterial activity against Gram-negative bacteria, indicating that *Bacillus altitudinis/pumilus* complex may only be effective against Gram-positive bacteria. Similar findings were reported in Brazil in 2021 where a *Bacillus altitudinis* isolate from wetland sediment showed no antibacterial activity against Gram-negative bacteria.^21^ The presence of the outer membrane in Gram-negative bacteria limits cellular permeability,^24^ and may thus result in poor diffusion of antimicrobial metabolites into the cytoplasmic membrane for potential inhibition of bacterial growth.^25^

### Limitations

Due to limited resources and funds, we were not able to identify the presumptive *Bacillus* species with negative antibacterial activity beyond the genus level. We also could not isolate, purify, or characterise the active metabolites from the *Bacillus altitudinis/pumilus* complex isolate with positive antibacterial activity.

### Conclusion

Our findings suggest that metabolites from soil-isolated *Bacillus altitudinis/pumilus* complex can be a potential source of antimicrobial agents with activity against Gram-positive bacteria, including MRSA and inducible clindamycin-resistant strains.
